# Seasonal and herbivore-induced dynamics of foliar glucosinolates in wild cabbage (*Brassica oleracea*)

**DOI:** 10.1007/s00049-018-0258-4

**Published:** 2018-05-10

**Authors:** Rieta Gols, Nicole M. van Dam, Michael Reichelt, Jonathan Gershenzon, Ciska E. Raaijmakers, James M. Bullock, Jeffrey A. Harvey

**Affiliations:** 1 Laboratory of Entomology, Wageningen University & Research, PO Box 16, 6700 AA Wageningen, The Netherlands; 2 German Centre for Integrative Biodiversity Research, Leipzig, Germany; 3 Max Planck Institute for Chemical Ecology, Jena, Germany; 4 Centre for Ecology and Hydrology, Wallingford, UK; 5 Department of Terrestrial Ecology, Netherlands Institute of Ecology, Wageningen, The Netherlands; 6 Department of Ecological Sciences, Section Animal Ecology, VU University Amsterdam, De Boelelaan 1085, 1081 HV Amsterdam, The Netherlands

**Keywords:** *Brassica oleracea*, Cabbage, Glucosinolates, Plant defence, Plant insect interactions, Secondary plant metabolites

## Abstract

Levels of plant secondary metabolites are not static and often change in relation to plant ontogeny. They also respond to abiotic and biotic changes in the environment, e.g., they often increase in response to biotic stress, such as herbivory. In contrast with short-lived annual plant species, especially those with growing periods of less than 2–3 months, investment in defensive compounds of vegetative tissues in biennial and perennial species may also vary over the course of an entire growing season. In garden experiments, we investigated the dynamics of secondary metabolites, i.e. glucosinolates (GSLs) in the perennial wild cabbage (*Brassica oleracea*), which was grown from seeds originating from three populations that differ in GSL chemistry. We compared temporal long-term dynamics of GSLs over the course of two growing seasons and short-term dynamics in response to herbivory by *Pieris rapae* caterpillars in a more controlled greenhouse experiment. Long-term dynamics differed for aliphatic GSLs (gradual increase from May to December) and indole GSLs (rapid increase until mid-summer after which concentrations decreased or stabilized). In spring, GSL levels in new shoots were similar to those found in the previous year. Short-term dynamics in response to herbivory primarily affected indole GSLs, which increased during the 2-week feeding period by *P. rapae*. Herbivore-induced changes in the concentrations of aliphatic GSLs were population-specific and their concentrations were found to increase in primarily one population only. We discuss our results considering the biology and ecology of wild cabbage.

**Electronic supplementary material:**

The online version of this article (10.1007/s00049-018-0258-4) contains supplementary material, which is available to authorized users.

## Introduction

In herbaceous plants, foliar chemical defences tend to increase within developmental stages, but also across the entire ontogenetic trajectory (Barton and Koricheva 2010). Often, concentrations of secondary metabolites vary over the growing season (Nelson et al. 1981; Velasco et al. 2007), which in short-lived (e.g. annual) plants may encompass their entire life cycle. In biennial and perennial species investment in foliar defensive compounds may not only vary within but also across seasons. Seasons are characterized by changes in abiotic factors such as light conditions (day length, shading) and temperature, which also affect secondary chemistry (Agerbirk et al. 2001; Gouinguene and Turlings 2002; Akula and Ravishankar 2011). Plants further respond to biotic factors such as pathogen infection and insect herbivory by increasing their levels of secondary metabolites, thereby minimising the investment in defence until it is necessary (Karban and Baldwin 1997). The production of secondary metabolites can also be constrained by biosynthetic and ecological costs (Hamilton et al. 2001; Strauss et al. 2002). Thus, levels of secondary metabolites in plants at a given time are the result of both genetic and environmental factors.

One well-studied group of secondary metabolites are glucosinolates (GSLs), which are characteristically produced by all plants in the Capparales including species in the cabbage and mustard family (Brassicaceae) (Fahey et al. 2001). This family includes important crop plant species such as various cultivars of cabbage (*Brassica oleracea*) and oil seed species (e.g. *B. napus, B. juncea*), as well as the model plant for genetic and molecular research, *Arabidopsis thaliana*. A good model system in the Brassicaceae for studying temporal variation in plant chemical defences is the wild cabbage, *B. oleracea* L. It is a perennial crucifer that grows naturally along the Atlantic coastlines of western Europe and is the ancestor of all cultivated cabbage varieties (Wichmann et al. 2008). The plant can live up to 10 years in the field and produces shoots that do not fall from the plant in autumn but which may remain attached to the stem of the plant until the next spring when new shoots are produced. Moreover, populations of wild cabbage growing in the south-west of England differ significantly in GSL composition and concentration (Mithen et al. 1995; Moyes and Raybould 2001; Gols et al. 2008). These differences in GSL composition among the populations correlate significantly with herbivore pressure (Newton et al. 2009, 2010). The responses to variation in the GSL profiles have been shown to be herbivore-specific, which may explain how diversity in GSL profiles is maintained in these populations (Newton et al. 2010). Moreover, when wild cabbage plants originating from different populations were grown together in plots in a garden experiment, colonization by herbivores and the amount of damage that they caused were not only determined by GSL chemistry of the focal plant but also by that of its neighbouring plants (Bustos-Segura et al. 2017).

**Fig. 1:**
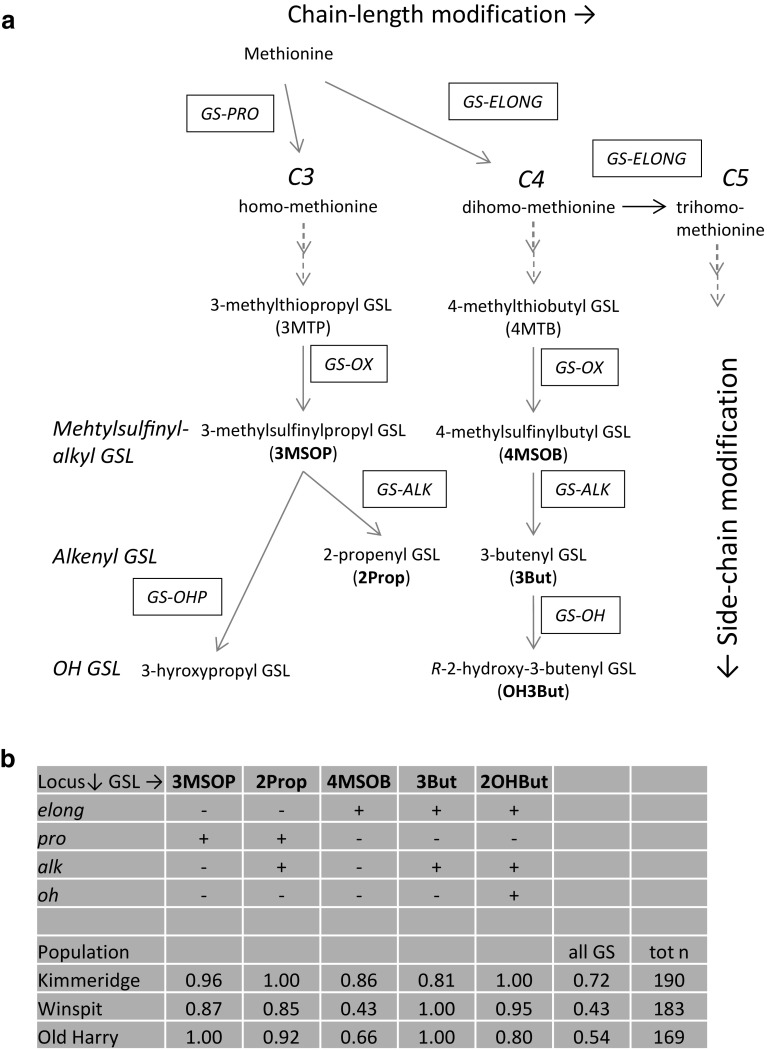
Biosynthesis of aliphatic glucosinolates (GSLs) **a** (modified according to Mithen et al. 1995) and **b** presence/absence of functional alleles at the four loci required for the biosynthesis of the specific GSLs found in leaves of the three wild cabbage populations (Winspit, Kimmeridge and Old Harry) used in this study. Abbreviated compounds in bold font are detected in the leaves of wild *B. oleracea*. In **b** GSL distributions are given as fractions of plants producing a specific GSL compound in the leaves with *n* the number of plants per population (data from Bustos-Segura et al. 2017)

However, in the studies above, GSLs were measured at single time points or were restricted to qualitative aspects of the GSL profile (i.e. the presence or absence of specific GSLs). GSLs have been reported to also exhibit seasonal variability (Agerbirk et al. 2001; Haribal and Renwick 2001; Velasco et al. 2007). Here, we investigated different temporal aspects of GSL dynamics, i.e. (1) short-term dynamics in response to herbivory and (2) long-term dynamics in relation to plant ontogeny and season in the perennial *B. oleracea*. Concentrations of GSLs were assessed in plants grown from seeds of wild cabbage that originated from three selected Dorset (UK) populations that have been demonstrated to differ qualitatively and quantitatively in their GSL profiles (Gols et al. 2008; Harvey et al. 2011). Variation in qualitative aliphatic GSL chemistry in *B. oleracea* is predominantly caused by differences in allele frequencies at four loci (Mithen et al. 1995) (Fig. 1). In this study, temporal variation in GSLs in relation to plant ontogeny were assessed in common garden experiments over a 1- and 2-year period. Using the same seed batches, we also investigated short-term foliar GSL dynamics in response to herbivory, i.e. inducibility of GSLs, in response to feeding damage inflicted by a specialist chewing herbivore *Pieris rapae* L. (Lepidoptera: Pieridae) that commonly feeds on these plants in the UK. The latter experiments were conducted in a greenhouse to control levels of herbivory.

## Materials and methods

### Plants and insects

Seeds of *B. oleracea* were collected from different individual plants (> 15) in three wild cabbage populations growing within 10–20 km of each other on chalk cliffs along the south coast of Great Britain, near Swanage in Dorset. The three wild populations were located at sites known as ‘Old Harry’ (‘OH’, 50°38′N, 1°55′W), ‘Kimmeridge’ (‘KIM’, 50°36′N, 2°07′W), and ‘Winspit’ (‘WIN’, 50°35′N, 2°02′W). These populations significantly differ in qualitative and quantitative GSL characteristics (Fig. 1, Harvey et al. 2011).

To measure foliar GSL dynamics in response to herbivory, we used caterpillars of the small cabbage white butterfly, *P. rapae* L. (Lepidoptera: Pieridae), which is a specialist of brassicaceous plant species and is frequently found feeding on the wild cabbage populations in England (Moyes et al. 2000; Newton et al. 2010). Larval feeding damage of this species produces irregular sized holes throughout the leaf. The caterpillars used in the experiments were obtained from the general insect rearing facility at Wageningen University (WU) where they have been reared on Brussels sprout plants (*B. oleracea* var. gemmifera cv. Cyrus) for many generations.

### Glucosinolate dynamics in leaves and seeds of field-grown plants (experiment 1)

In a previous study, it was demonstrated that aliphatic GSL concentrations in leaf tissues collected from plants growing naturally in Dorset and in a greenhouse correlated positively, although their absolute levels were lower in greenhouse-grown plants (Mithen et al. 1995). In a pilot study, we measured similar GSL concentrations in tissues collected from plants grown in sand and peat soil (supplementary data, Fig. S1). Based on these results, we predict that GSL concentrations measured in plants growing in a common garden in the Netherlands at the same latitude under similar climatological conditions as the wild cabbage plants in the UK would deviate little from those measured in plants growing at their original location in the UK.

In experiment 1a, GSL dynamics in leaf tissues were determined over a 2-year period. Seeds were germinated in moist peat soil and seedlings were transferred to a common garden in May 2006 in a plot near the Netherlands Institute of Ecology in Heteren. Seedlings were grown in nutrient enriched potting soil (Lentse potgrond no. 4, Lent, The Netherlands) in a layer ~ 30 cm deep on top of sand. Twelve seedlings from the three populations, 36 plants in total, were planted in alternating positions in two rows with a between-plant distance of one meter. Plants were watered when necessary. No additional fertilisation was applied, neither were the herbivores that colonized the plants removed. Leaf tissues were sampled for GSL analysis from each plant three times: in September 2006 in and in May and August of the following year. Individual plants were sampled by cutting one-third of the distal part of 6–10 fully expanded leaves of various ages. Leaves that had turned yellow were excluded from sampling. Collected leaf tissues were flashfrozen in liquid nitrogen immediately after sampling, pooled and stored at − 20 °C until GSL analysis. In 2008 most plants had few leaves and only produced flowers and seeds; therefore, leaf tissues were not collected in this year.

In an additional experiment (1b) conducted in 2012 we measured GSL dynamics within a growing season. Here we used plants of the KIM and OH population only. Seeds were germinated and seedlings were grown in the same soil as mentioned above. Seedlings were transferred to a garden plot adjacent to Wageningen University on May 23 and plants were sampled at June 15, July 12, August 23, October 4 and December 5 in 2012. We also sampled plants in May 2013 to confirm the results of the previous experiment. Plants were sampled only once: at the various time points leave tissues were collected from different plant individuals. Ten to 12 plants were sampled per population at each time point. Ten leaf discs (*Ø* = 1.0 cm) were punched from various leaves using a cork borer, flashfrozen in liquid nitrogen immediately after sampling, pooled and stored at − 20 °C until analysis.

### Glucosinolate dynamics in response to herbivory in greenhouse-grown plants (experiment 2)

In a second experiment we measured GSL dynamics in response to herbivory in a greenhouse were herbivory could be controlled. Seeds were germinated and seedlings were transferred to 2.1-l pots filled with peat soil (Lentse potgrond #4). Plants were watered daily. Greenhouse conditions were set at 18–25 °C, 40–80% r.h. and a photoperiod of at least 16 h. If the light dropped below 500 µmol photons m^−2^ s^−1^ during the 16-h photoperiod, supplementary illumination was applied (SON-T). When the plants were 4 weeks old, they were fertilized once a week with ‘Kristallon blauw’ (N:P:K:micro nutrients as 19:6:20:4) at 2.5 mg l^−1^, which was applied to the soil. Plants were 6 weeks old when they were used in experiments.

To investigate the temporal changes in GSL levels in response to herbivory, GSLs were measured at four time points; 0, 4, 8 and, 16 days following infestation with *P. rapae* caterpillars. Per population, 9–11 plants were each infested with 10 first instar *P. rapae* caterpillars divided over three leaves. Caterpillars were free to move and feed within a plant. Sampling consisted of punching leaf discs (*Ø* = 1.7 cm), one per leaf, from 5 to 6 fully unfolded leaves. The leaf discs were pooled per plant, flashfrozen in liquid nitrogen immediately after sampling and stored at − 20 °C. The same plants were sampled repeatedly during the induction period.

In an additional experiment using only two populations, WIN and OH with ten plants per population, we determined the effect of repeated mechanical damage on GSL dynamics. Mechanical damage was inflicted on day 0, 4 and 8, and 16 by punching holes in 5–6 leaves, two holes per leaf, using a cork borer (*Ø* = 1.0 cm). The removed tissues were pooled per plant and used for GSL analysis. For comparison with the previous experiment with herbivores, extra sets of 8–10 control and herbivore-exposed plants were sampled at day 16 only. As *P. rapae* was no longer available, plants were infested with six neonate *P. brassicae* caterpillars that were introduced onto the plant at day 0.

### Glucosinolate analysis

Tissues collected in experiment 1a and 1b were extracted and analysed as follows: GSLs were cold-extracted with 1.00 ml of 80% methanol solution containing 50.0 µM intact 4-hydroxybenzyl GSL as internal standard, desulfated with arylsulfatase (Sigma-Aldrich) on a DEAE Sephadex A 25 column. The eluted desulfoglucosinolates were separated using high performance liquid chromatography (Agilent 1100 HPLC system, Agilent Technologies, Waldbronn, Germany) on a reversed phase C-18 column (Chromolith Performance RP18e, 100 × 4.6 mm, Merck, Darmstadt Germany) with an water-acetonitrile gradient (0–3% acetonitrile from 0 to 3 min, 3–23% acetonitrile from 3 to 11 min, 23–33% acetonitrile from 11 to 13 min, followed by a washing cycle; flow 1 ml min^−1^). Detection was performed with a photodiode array detector and peaks were integrated at 229 nm. For peak identification, some samples were run on an LC-IonTrap-MS-system to determine [M–H]− in negative mode. *R*-2-hydroxy-3-butenenyl GSL was identified based on its retention time determined in rapeseed samples.

GSLs in tissues obtained in experiment 2 were extracted and analysed in a different laboratory using a slightly different method (van Dam et al. 2004). GSLs were extracted twice with 1 ml of boiling 70% methanol solution, desulfated with arylsulfatase (Sigma-Aldrich) on a DEAE Sephadex A 25 column. The compounds were separated on a DIONEX summit HPLC, (DIONEX, Sunnyvale, CA, USA) on a reversed phase C-18 column (Alltima C-18, 150 × 4.6 mm, 3 µm, Alltech, Deerfield, IL, USA) with an acetonitrile–water gradient (2–35% acetonitrile from 0 to 30 min; flow 0.75 ml min^−1^). Detection was performed with a photodiode array detector and peaks were integrated at 229 nm. 2-Propenyl GSL (ACROS, New Jersey, USA) was used as an external standard.

To calculate the concentrations of the different types of GSLs in both analyses, we used the generally accepted relative response factors (Wathelet et al. 2004) for detection at 229 nm in relation to the respective standards. GSL compounds were classified based on their amino acid origin as indole GSLs (derived from tryptophan) and aliphatic GSLs (derived from methionine) (Halkier and Gershenzon 2006). Small amounts of 2-phenylethyl GSL (abbreviated to 2PE), the sole GSL derived from phenylanaline, were detected as well, but they were only consistently found in the WIN population. We further divided the aliphatic GSLs in subgroups according to their side-chain modification (Fig. 1a). Aliphatic GSL compounds were abbreviated as depicted in Fig. 1. Indole GSLs were abbreviated as: I3M = indolyl-3-methyl GSL; 1MOI3M = 1-methoxyindolyl-3-methyl GSL; 4MOI3M = 4-methoxyindolyl-3-methyl GSL; 4OHI3M = 4-hydroxyindolyl-3-methyl GSL.

### Statistics

GSL dynamics in plants grown in a common garden (experiment 1a) were measured repeatedly on the same plant and were analysed using a mixed model analysis of variance with population, sampling time, and their interaction as fixed factors and plant individual as a random variable. The effects in the model were based on restricted likelihood estimation (REML) using SAS 9.3 (SAS Institute Inc., Cary, NC, USA), GSL concentrations were log-transformed to meet assumptions of normality and homoscedasticity.

Multivariate statistics (i.e. principal components analysis, PCA) were used to separate the plants in relation to population origin and sampling date based on their foliar GSL concentrations. We used the projection to latent structures (PLS by means of partial least squares projections) extension of the program (SIMCA 15.0, Umetrics, Umeå, Sweden), which relates variables in the *X* matrix (GSLs) to variables in the *Y* matrix (population classes and sampling times). The program’s cross validation procedure evaluates the significance of each additional component (starting with none) by comparing the goodness of (*R*^2^) and the predictive value (*Q*^2^) of the extended model with that of the reduced model (Eriksson et al. 2006).

We used orthogonal projections to latent structures (OPLS) to reveal linear time dependent changes in GSL concentration for each population separately: (1) over the growing season, (2) in response to *P. rapae* feeding duration and (3) In response to repeated mechanical damage. OPLS allows for separation of the systematic variation into predictive (i.e. explained by herbivory) and orthogonal variation. In these analyses time was included as a quantitative variable.

In all of these analyses, per sample, concentrations of the individual GSL compounds, total aliphatic and total indole GSLs, grand totals and % aliphatic GSLs served as variables in the model. Data were log-transformed, mean-centred and scaled to unit variance before they were subjected to the analysis.

## Results and discussion

### Seasonal dynamics in glucosinolates concentrations

**Fig. 2:**
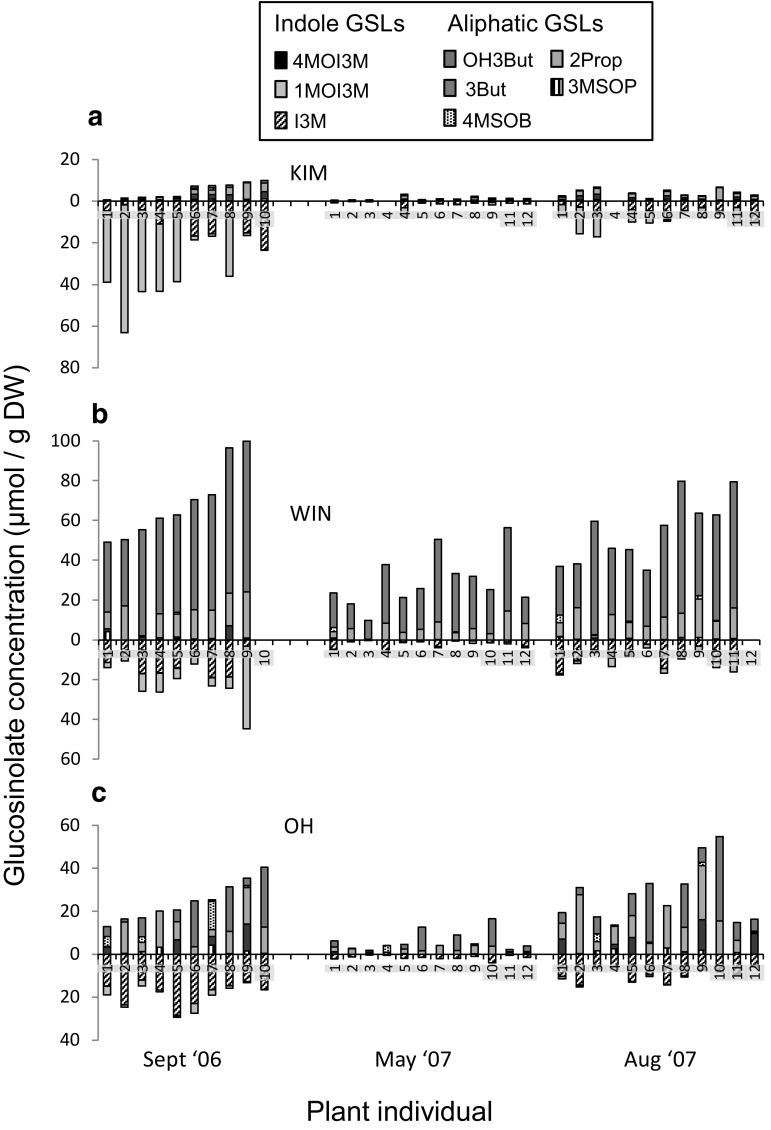
Foliar glucosinolate (GSL) concentrations measured repeatedly in the same plant individual during 2 years of growth in three wild cabbage populations, **a** KIM, **b** WIN and **c** OH, originating in Dorset, England and grown in a common garden. Concentrations above the *x*-axis depict aliphatic GSL concentrations, those below the *x*-axis depict indole GSLs. Pants were sampled in September 2006 (first year of growth), and in May and August of the following year. For the full names of the GSL compounds see Fig. 1 and the “Glucosinolate analysis” section

**Fig. 3:**
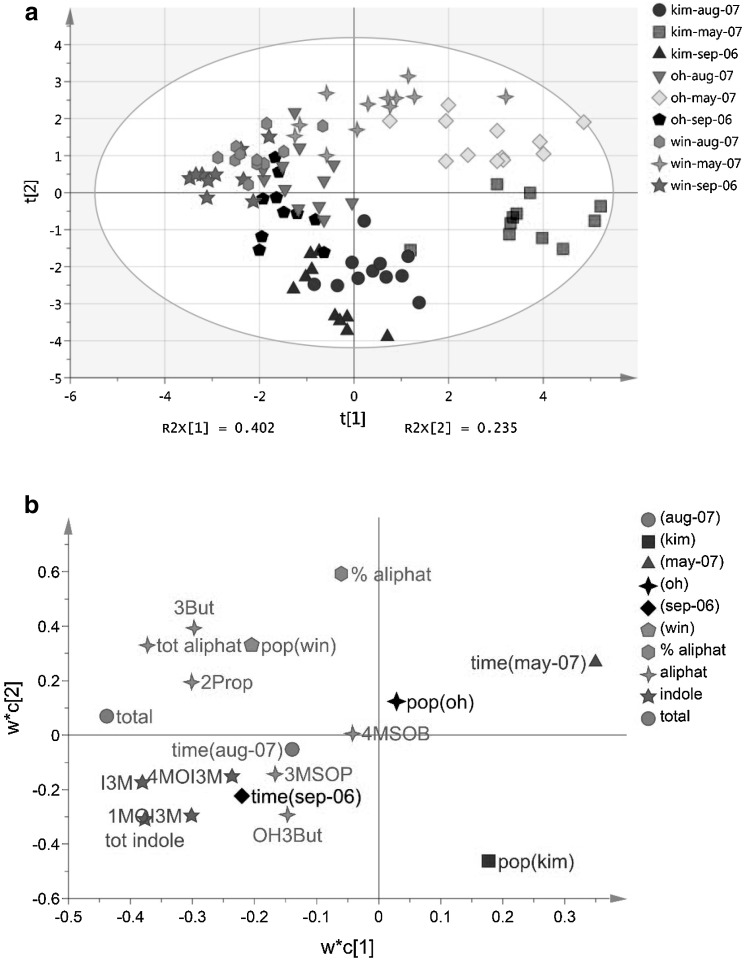
Multivariate analysis (PLS by means of partial least squares projections) of glucosinolates (GSLs) in the leaves. GSLs were measured in plants from three wild cabbage populations KIM (blue symbols), OH (green symbols) and WIN (yellow–red symbols) originating in Dorset, England that were grown in a common garden and of which leaves were sampled in September 2006 (light coloured symbols) and in May and August of the following year (darker coloured symbols). The score plot **a** visualises the structure of the samples according to the first two latent variables. *R*^2^*X*[1] and *R*^2^*X*[2] depict the variance fraction explained by the latent variables. The ellipse in the score plots defines the Hotelling’s T2 confidence region and provides a 95% confidence interval for the observations. The loading plot **b** defines the orientation of the PC planes with the original variables. The full names of the GSL compounds are given in Fig. 1 and the “Glucosinolate analysis” section. *tot aliphat* totals of aliphatic GSLs, *tot indole* total of indole GSLs, *total* grand totals of GSLs, *% aliphat* percentage aliphatic of total. Model statistics for foliar GSLs with three significant components: *R*^2^*X* = 0.74, *Q*^2^ = 0.49. (Color figure online)

The five different aliphatic and three different indole GSLs that were detected in the leaf tissues were present in all three plant populations (Figs. 2, 3). However, foliar concentrations of the different GSLs and classes of GSLs varied considerably among the three populations and these also depended on the time of sampling (Table 1, Figs. 2, 3a). Population-specific differences in foliar GSLs were similar as described before for these populations (e.g. Harvey et al. 2011). Briefly, leaf tissues of KIM plants contain relatively high concentrations of indole GSLs (85% of the total GSL content in Fig. 2a), whereas OH and WIN leaf tissues contain, relatively and absolutely, high concentrations of aliphatic GSLs [contributing on average 71 (OH) and 85% (WIN) to the total foliar GSL content in Fig. 2b, c]. Overall GSL concentrations are the highest in the leaves of WIN plants. PLS multivariate analysis separated the plants according to their sampling date and population origin (Fig. 3a). GSL concentrations, especially those of I3M, 1MOI3M, 2Prop and 3But, were much lower in leaf tissues sampled in May 2007 than in tissues sampled in September 2006 and August 2007 (Figs. 2, 3a). The plants grow new foliage in spring and drop the old leaves. The GSL dynamics in the newly grown leaves appear to follow the same seasonal patterns as in the previous year.

**Table 1:** Significance levels of statistical tests (“Materials and methods”) on seasonal long-term (across seasons) effects on the dynamics of glucosinolate (GSL) concentrations in *Brassica oleracea* leaves

Compound	Pop	Time	Time × pop
*Aliphatic*			
C3			
3MTP	–	–	–
3MSOP	–	–	–
2-Prop	< *0.001*	< *0.001*	0.06
C4			
4MTP	–	–	–
4MSOB	–	–	–
3But	< *0.001*	< *0.001*	< *0.001*
OH3But	0.21	< *0.001*	0.33
Total aliphat	< *0.001*	< *0.001*	*0.002*
*Indole*			
I3M	< *0.02*	< *0.001*	0.27
1MOI3M	*0.002*	< *0.003*	0.006
4MOI3M	< *0.001*	< *0.001*	0.88
Total indole	0.34	< *0.001*	0.06

Italics depict significant effects. Seeds originated from three populations (pop effect), WIN, KIM and OH, in Dorset, England. GSLs were classified as aliphatic with variable change length (C3 or C4) or indolic. For the full names of the GSL compounds, see Fig. 1 and the “Glucosinolate analysis” section

For the six most dominant compounds, both the effect of sampling time and population were significant with the exception of OH3But for which the population effect was not significant (Table 1). Interestingly, the population-sampling interaction term was significant for 2Prop and 3But, but not for any of the indole GSLs. This means that the seasonal effect on indole GSLs is similar in the three populations (low in spring, high in autumn), but that it differentially affected the alkenyl GSLs 2Prop and 3But depending on population origin.

**Fig. 4:**
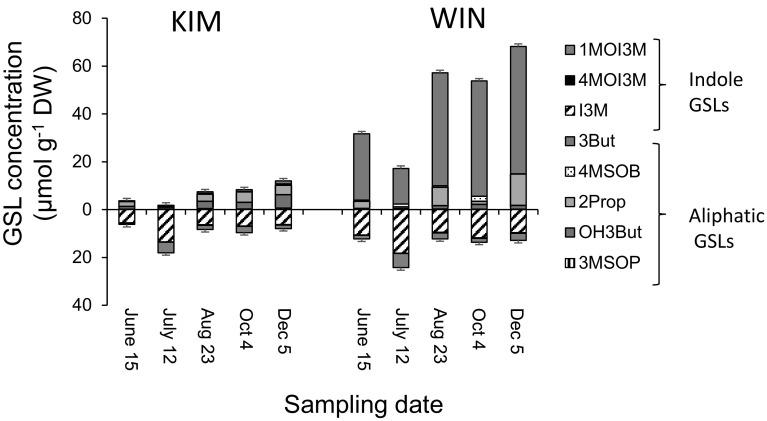
Within season dynamics of aliphatic (above the *x*-axis) and indole GSLs (below the *x*-axis) in leaf tissues in plants originating from the KIM and WIN population. Plants were grown in a common garden in 2012 and sampled at June 15, July 12, August 23, October 4 and December 5. The full names of the GSL compounds are given in Fig. 1 and the “Glucosinolate analysis” section. Error bars depict the mean standard error of the total aliphatic and indole GSL concentrations, respectively (*n* = 10–12)

**Fig. 5:**
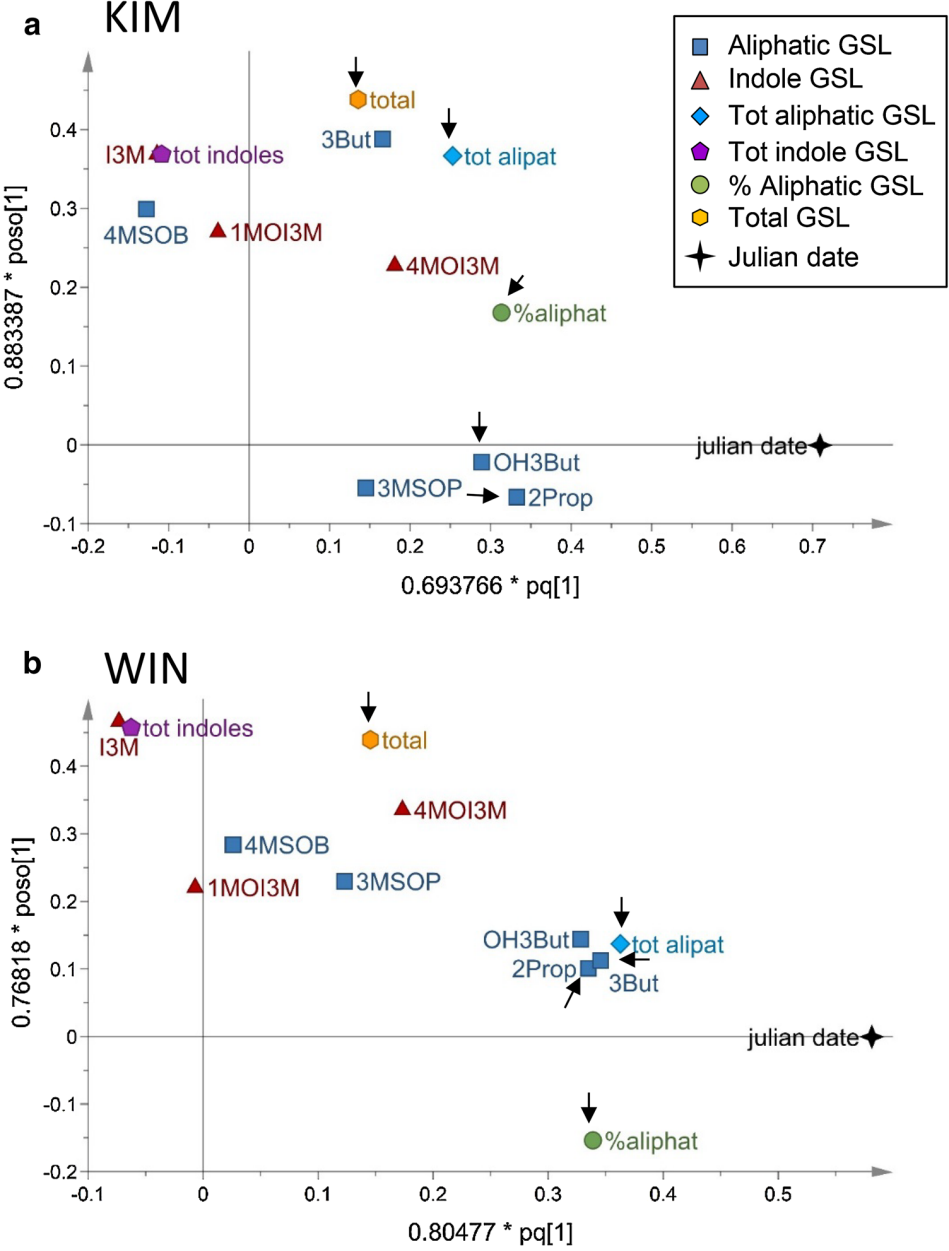
Multivariate analysis of within year glucosinolate dynamics in leaf tissues in plants originating from two wild cabbage populations, **a** KIM, **b** WIN in Dorset, England. GSL data presented in Fig. 4 were subjected to OPLS by means of partial least squares projections. The horizontal axis coincides with day of the year (Julian data) from left to right. The full names of aliphatic GSLs compounds are given in Fig. 1 and the “Glucosinolate analysis” section. *tot aliphat* totals of aliphatic GSLs, *tot indole* total of indole GSLs, *total* grand totals of GSLs, *% aliphat* percentage aliphatic of total. Arrows point at variables of which the correlation coefficient with time is significantly different from 0 and at the same time these variables contributed significantly to the separation of samples in relation to time based on model variable importance values (VIP). Variables with VIP > 1 are highly influential (Eriksson et al. 2006). Model statistics for KIM: overall significance statistical model *F*_2,56_ = 15.9, *P* < 0.001; OPLS predictive statistics, *R*^2^*X* = 0.417, *R*^2^*Y* = 0.398, *Q*^2^ = 0.362; for WIN: overall model significance, *F*_2,56_ = 12.2, *P* < 0.001; OPLS predictive statistics *R*^2^*X* = 0.353, *R*^2^*Y* = 0.364, *Q*^2^ = 0.303

In experiment 1b, within-season GSL dynamics were followed in more detail for WIN and KIM plants. The increase from May to December was statistically significant for the aliphatic GSLs, 3But, OH3But and 2Prop and this effect was stronger in WIN than in KIM plants (Figs. 4, 5). The seasonal dynamics of the indole GSLs were more idiosyncratic. For example, concentrations of I3M and to a lesser extent for 1MOI3M peaked in July (Fig, 4), whereas concentrations of 4MOI3M increased continuously (Fig. 5). In both plant populations, GSL concentrations did not differ between plants sampled in June 2012 and May 2013, with the exception of OH3But in WIN plants, of which concentrations were marginally higher in May 2013 than in June 2012 (*F*_1,28_ = 4.11, *P* = 0.052). In addition to abiotic factors, natural infestations with herbivores are likely to have confounded the effects that can strictly be attributed to plant ontogeny. Nevertheless, our data show that changes in glucosinolate chemistry over the season can be quite dramatic depending on plant population and are GSL-class specific.

### Glucosinolates dynamics in response to herbivory

**Fig. 6:**
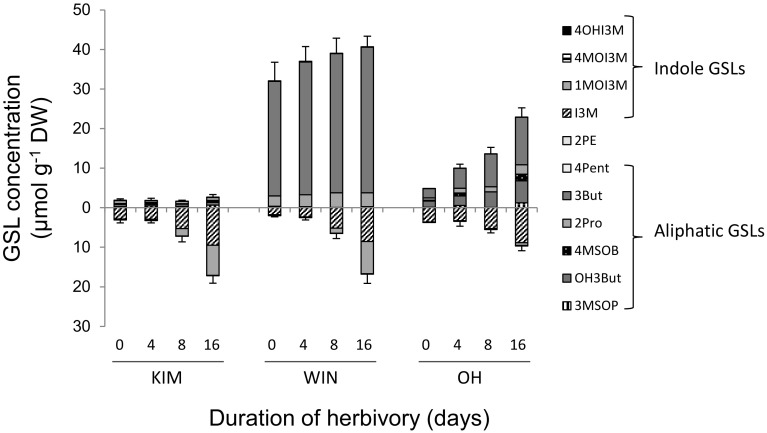
Glucosinolate dynamics in response to *Pieris rapae* feeding in leaf tissues of plants originating from three wild cabbage populations (KIM, WIN and OH). Concentrations above the *x*-axis depict aliphatic GSL concentrations, those below the *x*-axis depict indole GSLs. Error bars depict the mean standard error of the total aliphatic and indole GSL concentrations, respectively (*n* = 9–11). Pants were grown in a greenhouse and sampled at *t* = 0, 4, 8 and 16 days following introduction of ten first instar *P. rapae* caterpillars. The full names of the GSL compounds are given in Fig. 1 and the “Glucosinolate analysis” section (*4Pent* 4 pentenyl GSL)

**Fig. 7:**
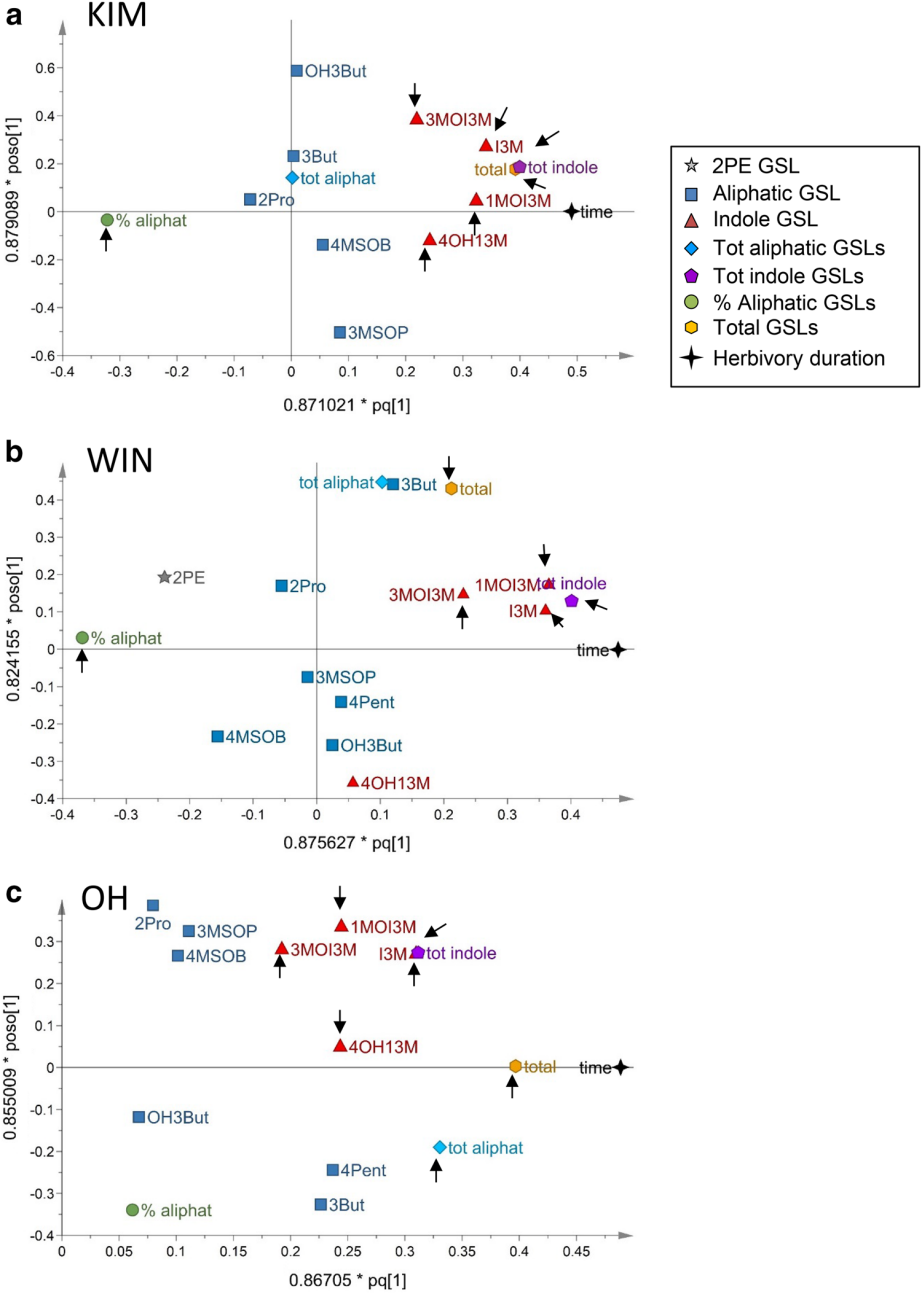
Multivariate analysis of glucosinolate dynamics in leaf tissues in response to feeding by *Pieris rapae* caterpillars in plants originating from three wild cabbage populations, **a** KIM, **b** OH, and **c** WIN in Dorset, England. GSL data were subjected to OPLS by means of partial least squares projections. The horizontal axis coincides with increased duration of feeding from left to right. Plants were grown in a greenhouse and sampled repeatedly at *t* = 0, 4, 8 and 16 days since the introduction of ten first instar *P. rapae* caterpillars. The full names of aliphatic GSLs compounds are given in Fig. 1 and the “Glucosinolate analysis” section. *tot aliphat* totals of aliphatic GSLs, *tot indole* total of indole GSLs, *total* grand totals of GSLs, *% aliphat* percentage aliphatic of total, *4Pent* 4-pentenyl GSL. Arrows point at variables of which the correlation coefficient with time is significantly different from 0 and at the same time these variables contributed significantly to the separation of samples in relation to time based on model variable importance values (VIP). Variables with VIP > 1 are highly influential (Eriksson et al. 2006). Model statistics for KIM: overall significance statistical model *F*_2,36_ = 33.0, *P* < 0.001, OPLS predictive statistics *R*^2^*X* = 0.347, *R*^2^*Y* = 0.712, *Q*^2^ = 0.647; for WIN: overall model significance, *F*_2,40_ = 49.3, *P* < 0.001; OPLS predictive statistics *R*^2^*X* = 0.317, *R*^2^*Y* = 0.743, *Q*^2^ = 0.711; for OH: overall model significance, *F*_2,32_ = 35.1, *P* < 0.001; OPLS predictive statistics *R*^2^*X* = 0.325, *R*^2^*Y* = 0.719, *Q*^2^ = 0.687

To tease apart the effect of herbivory from other effects, short-term GSL dynamics were followed in plants grown under greenhouse conditions were herbivory could be controlled. All indole GSLs increased in response to herbivory but not to the same extent and this also depended on the population; I3M and 1MOI3M responded the strongest to *P. rapae* feeding and changes in concentrations of 1MO3IM were much pronounced in KIM and WIN than in OH plants (Figs. 6, 7). In WIN and KIM plants, concentrations of 1MOI3M were more than 40 times higher after 16 days of *P. rapae* feeding compared to the levels measured at *t* = 0 when the caterpillars were introduced. Herbivore-induced changes in aliphatic GSL were only found in OH plants. In this population, aliphatic GSLs tended to increase when exposed to *P. rapae* feeding (Figs. 6, 7). The only compound that decreased in response to herbivory was 2PE in WIN plants that produced this compounds consistently albeit in very low concentrations. This compound is more common in root tissues of *Brassica* species in which it is often the dominant GSL and tends to increase in response to root herbivory (van Dam et al. 2009). In root tissues of the populations used in this study, it can contribute 25–40% to the total GSL content (van Geem et al. 2016).

The results described above may have been somewhat inflated by the mechanical damage caused by the repeated sampling of the same plant, which has been shown to induce primarily the indole GSLs in *Brassica* species (Bodnaryk 1992; Pontoppidan et al. 2005). However, the additional experiment in which we measured the changes in GSL concentrations in response to mechanical damage, revealed that only the aliphatic GSLs 3But and OH3But significantly increased in response to mechanical damage, whereas concentrations of the indole GSLs were not affected (supplementary data, Figs. S2–3). The effects of mechanical damage on GSL concentrations were relatively small as the statistical model was only significant for WIN and not for OH plants (Fig. S3). These results suggest that the changes in indole GSL concentrations are caused by caterpillar feeding and not by mechanical damage.

Among the different classes, indole GSLs appear to be the most responsive to herbivory (Agerbirk et al. 2009; Hopkins et al. 2009; Textor and Gershenzon 2009), whereas herbivore-induced changes in aliphatic GSLs appear to be more idiosyncratic (van Dam et al. 2009). The increase of indole GSLs from May to August in the field-grown plants may reflect herbivore pressure which is also highest during this period (Bustos-Segura et al. 2017). In nature, levels of inducible indole GSLs in wild *B. oleracea*, which are much higher than has been thus far reported in any other brassicaceous species, may play an important role against specialist herbivores such as *P. rapae, Plutella xylostella* and *Athalia rosae* (Gols et al. 2008; Harvey et al. 2011; Abdalsamee and Müller 2012). These herbivore species are specialist feeders on plants containing GSLs and are able to effectively circumvent exposure to toxic aliphatic and aromatic GSL breakdown products (Müller et al. 2001; Ratzka et al. 2002; Wittstock et al. 2004). The effects of high concentrations of indole GSLs that are present in some wild *B. oleracea* populations on the performance of specialist insect herbivores merits further study.

### Seasonal and herbivore-induced differences in glucosinolate dynamics among the cabbage populations

GSL concentrations within and between classes varied considerably among the populations and are consistent with previously reported results (Mithen et al. 1995; Moyes et al. 2000; Gols et al. 2008). In addition, the dynamics of GSLs varied as well among the populations, both over the season and in response to herbivory. It is not clear to what extent GSL-related differences among the relatively small populations of wild *B. oleracea* plants growing along the English coastlines are the result of variable selection from abiotic and biotic factors such as insect herbivores and pathogens. Wichmann et al. (2008) demonstrated that the spatial distribution of cabbage populations in the county of Dorset has changed little over at least the past 70 years. Each of the populations used in this study is exposed to significant variation in abiotic and perhaps biotic conditions. For example, the KIM population grows on an exposed cliff top and is subject to strong prevailing south-westerly winds, whereas the WIN population grows in a sheltered cove that only is exposed to gentle southerly winds. OH plants, on the other hand, also grow in an exposed site, but the cliffs are facing east and it is thus to some extent sheltered from prevailing south-westerly winds. Wind exposure may affect certain plant traits directly, but it may also affect the ability of herbivores to find and exploit plants, thus influencing selection exerted by these herbivores on plant defence traits. This may explain why KIM plants largely rely on indole GSL defences that are only produced upon herbivory and, thus, infest in defence when under attack. In the WIN and OH population, herbivory is most likely more predictable and, therefore, plants also have high levels of constitutive aliphatic GSL defences that increase with aging of the foliage. Newton et al. (2010) observed temporal consistencies in herbivore distributions among GSL genotypes across and within 12 wild cabbage populations growing along a linear coastline gradient in Dorset, UK. GSL genotype differentially affected several herbivore species which could explain how diversity in GSL chemistry is maintained in brassicaceous plant species (Lankau 2007; Bidart-Bouzat and Kliebenstein 2008; Burow et al. 2010; Newton et al. 2010). Moreover, a recent study showed that increased variation in GSL chemistry among neighbouring plants correlated positively with herbivore diversity and negatively with plant damage (Bustos-Segura et al. 2017).

Longer-lived perennial plants, such as wild cabbage, which retain their foliage over winter and grow in stable habitats over extended time frames, are important to consider when studying the evolution, maintenance and genetic variation in traits related to resistance against insects. The interaction between GSLs and insects, with a few exceptions, is often studied in (short-lived) annual plants such as *A. thaliana* and some *Brassica* and *Sinapis* species, as well as cultivated *Brassica* species, which have been exposed to artificial selection (Gols and Harvey 2009; Hopkins et al. 2009; Textor and Gershenzon 2009). Wild cabbage grows in populations that are very stable (Wichmann et al. 2008) and individual plants can live for up to 10 years in the wild. In this situation, selection pressure from antagonists may be much more predictable as the plants remain in situ within for many years. By contrast, many annuals, such as the ‘model’ species *A. thaliana*, are strongly r-selected (e.g. trade off resources for rapid growth against reduced defence) have very short-life cycles and tend to ‘move around’ to different early successional sites from year to year. In these situations selection pressures from insects and other antagonists are often highly stochastic and thus less predictable. Therefore, the mechanisms maintaining genetic variation and the phenotypic expression in defence traits, such as GSLs in brassicaceous plant species, may differ profoundly in annual and perennial plant species. A broad interspecific and inter-population analysis of quantitative variation in GSL in the Brassicaceae might confirm the broader applicability of generalisations of defence expression in annual and perennial plants.

## Electronic supplementary material

Below is the link to the electronic supplementary material.

Supplementary material 1 (DOCX 382 KB)
