# Supplementary material for: Seasonal and herbivore-induced dynamics of foliar glucosinolates in wild cabbage (Brassica oleracea)
Source: Chemoecology. 2018 May 10;28(3):77–89. doi: 10.1007/s00049-018-0258-4 (PMC5988764; doi:10.1007/s00049-018-0258-4)
Supplement: Supplementary file 1 — Supplementary material 1 (DOCX 382 KB) [file 49_2018_258_MOESM1_ESM.docx]

Figure S1: Shoot glucosinolate concentrations (mean + SE, n=4-5) in wild, 6-wk old *Brassica oleracea* plants grown from seeds originating from three populations (KIM, OH and WIN) in Dorset, England either on sand (A) or peat soil (B). Half of the plants from each population was induced with *Plutella xylostella* (PLUT), whereas the other half was left undamaged (CON). Different letters over the bars indicate significant differences (P < 0.05) in total glucosinolate levels between the bars within each panel (Tukey HSD multiple comparisons among means).

Figure S2. Glucosinolate (GSL) dynamics in response to mechanical damage in leaf tissues of plants originating from two wild cabbage populations WIN and OH. Concentrations above the x-axis depict aliphatic GSL concentrations, those below the x-axis depict indole GSLs. Error bars depict the mean standard error of the total aliphatic and indole GSL concentrations, respectively. Ten pants of each population were grown in a greenhouse and sampled at t=0, 4, 8 and 16 days. Mechanical damage was inflicted by punching holes in 5 to 6 leaves, two holes per leaf, using a cork borer (Ø=1.0 cm) on day 0, 4 and 8 and 16. The removed tissues were pooled per plant and used for GSL analysis. For comparison, additional sets of 8-10 control (Cont) and herbivore-exposed (Induced) plants were sampled at day 16 only. In the Induced treatment, plants were infested with six neonate *P. brassicae* at day 0. Abbreviations glucosinolates (GSLs): 1MOI3M = 1 methoxyindolyl-3-methyl GSL; 4MOI3M = 4-methoxyindolyl-3-methyl GSL, I3M = indolyl-3-methyl GSL,3But = 3-butenyl GSL, 2Prop = 2-propenyl GSL, 4MSOB = 4-methylsulfinylbutyl GSL, OH3But = *R*-2-hydroxy-3-butenyl GSL; 3MSOP = 3-methylsulfinylpropyl GSL.

Figure S3. Multivariate analysis of foliar glucosinolate dynamics in response to repeated mechanical damage inflicted on day 0, 4, 8, and 16 (see Fig. S2) in plants originating from two wild cabbage populations, WIN and OH in Dorset, England. GSL data were subjected to Orhogonal Projection to Latent Structures by means of partial least squares projections. The horizontal axis coincides with time from left to right. Plants were grown in a greenhouse. For the full names of the abbreviated GSLs see Fig. S2. Tot aliphat = totals of aliphatic GSLs; tot indole = total of indole GSLs; total = totals of GSLs, % aliphat = percentage aliphatic of total. Arrows point at variables of which the correlation coefficient with time is significantly different from 0 and at the same time these variables contributed significantly to the separation of samples in relation to time based on model variable importance values (VIP). Variables with VIP>1 are highly influential ([Eriksson et al. 2006](#_ENREF_10)). Model statistics for WIN: overall significance statistical model *F*_2,37_ = 18.7, *P*<0.001, OPLS predictive statistics, *R*^2^*X* = 0.281, *R*^2^*Y* = 0.585, *Q*^2^ = 0.502; for OH: overall model significance *F*_2,37_ = 0, *P*=1, OPLS predictive statistics, *R*^2^*X* = 0.287, *R*^2^*Y* = 0. 125, *Q*^2^ = -0.011.
